# Improved speech intelligibility in the presence of congruent vibrotactile speech input

**DOI:** 10.1038/s41598-023-48893-w

**Published:** 2023-12-19

**Authors:** Alina Schulte, Jeremy Marozeau, Anna Ruhe, Andreas Büchner, Andrej Kral, Hamish Innes-Brown

**Affiliations:** 1 Department of Experimental Otology of the Clinics of Otolaryngology, Hannover Medical School, Hannover, Germany; 2 Eriksholm Research Center, Oticon A/S, Snekkersten, Denmark; 3 Music and Cochlear Implants Lab, Department of Health Technology, Technical University Denmark, Kongens Lyngby, Denmark; 4 Hearing Systems Section, Department of Health Technology, Technical University of Denmark, Kongens Lyngby, Denmark

**Keywords:** Language, Auditory system, Perception, Sensory processing

## Abstract

Vibrotactile stimulation is believed to enhance auditory speech perception, offering potential benefits for cochlear implant (CI) users who may utilize compensatory sensory strategies. Our study advances previous research by directly comparing tactile speech intelligibility enhancements in normal-hearing (NH) and CI participants, using the same paradigm. Moreover, we assessed tactile enhancement considering stimulus non-specific, excitatory effects through an incongruent audio-tactile control condition that did not contain any speech-relevant information. In addition to this *incongruent* audio-tactile condition, we presented sentences in an *auditory only* and a *congruent* audio-tactile condition, with the *congruent* tactile stimulus providing low-frequency envelope information via a vibrating probe on the index fingertip. The study involved 23 NH listeners and 14 CI users. In both groups, significant tactile enhancements were observed for *congruent* tactile stimuli (5.3% for NH and 5.4% for CI participants), but not for *incongruent* tactile stimulation. These findings replicate previously observed tactile enhancement effects. Juxtaposing our study with previous research, the informational content of the tactile stimulus emerges as a modulator of intelligibility: Generally, congruent stimuli *enhanced*, non-matching tactile stimuli *reduced,* and neutral stimuli did *not change* test outcomes. We conclude that the temporal cues provided by congruent vibrotactile stimuli may aid in parsing continuous speech signals into syllables and words, consequently leading to the observed improvements in intelligibility.

## Introduction

Cochlear implants (CIs) have revolutionized the therapy of hearing impairment with a substantial increase in speech intelligibility in the vast majority of CI recipients^1,2^. However, some CI recipients experience difficulties understanding speech, particularly in the presence of competing sounds^3,4^. Not all recipients benefit from CIs, highlighting the need to explore approaches that could further enhance speech perception through multisensory inputs^5^.

The view of the speaker's face significantly enhances speech perception^6–9^. The principles underlying this multimodal benefit were studied in single neuron recordings in the feline superior colliculus^10^, where firing rates in response to temporally and spatially aligned multimodal stimuli were found to be larger than the sum of the unimodal responses. This supra-additivity increased when the unimodal responses alone were close to the detection threshold (principle of inverse effectiveness (PoIE), see also^11–14^). Parallels between single-cell recordings and human behavioral studies have been shown both for simple detection tasks^15–19^ and for speech understanding^20,21^. Yet, the rules of multisensory integration (MSI) have not been reliably observed in behavioral measures^22^ with their evidence often contingent on the statistical method chosen^13,23,24^.

The PoIE suggests a parsimonious explanation for why CI patients may take better advantage of multimodal cues than those with good hearing. Nonetheless, studies about MSI abilities in CI users have provided mixed results. Most likely influenced by the specific experimental paradigm and variability of the onset of deafness and age of implantation within and between investigated samples, studies have reported *reduced*^25^, *indifferent*^26–28^ and *enhanced*^29,30^ MSI abilities in deaf individuals with CIs. Sensory experience, especially early in life, is key for the development of MSI abilities^5,31^. This explains findings on lower multisensory gain in congenitally deaf CI users compared to late-deaf CI recipients^32^ and early- versus late-implanted pediatric CI recipients^33^.

Not only visual, but also tactile input can facilitate speech perception^34^. Perceptual benefits with tactile aids (e.g. TickleTalker^35^) have been explored since the 1920s^36–39^. However, with the advances in CI technology, tactile aids have no longer received much attention. Only in recent years, with stagnation in further CI improvements in combination with readily available haptic wearables at low cost, the potential of audio-tactile speech was rediscovered^40–42^.

Recent research by Huang et al.^41^ explored the use of vibrotactile stimuli that mimic residual low-frequency information in the form of the fundamental frequency (f0) contour often inaccessible to CIs. They found a tactile advantage for speech understanding of 2.2 dB in the speech reception threshold (SRT), which is comparable to the improvement obtained from combined electric-acoustic hearing^43,44^.

Instead of the f0 contour^41,45^, other studies have used envelope information derived from low-frequency bands of the noisy^42,46^ or clear^47^ speech signal, or sparse vibrotactile pulses with the syllabic rhythm^48^ (based on the finding that neural entrainment in the theta range modulated speech-in-noise perception^49^). Depending on the participant group auditory stimuli have been presented as either clear or vocoded speech in a multi-talker^42,45–47,50^ or unmodulated speech-shaped noise background^41^. Irrespective of the exact approach, most publications have shown better performance in audio-tactile conditions compared to audio alone when speech was presented in noise to target intelligibility levels around 50%. The largest enhancements were reported by Cieśla et al.^45^, with an immediate improvement of 6 dB in SRT in normal-hearing (NH) participants presented with non-native vocoded speech and tactile vibration on the fingertips. In native NH English speakers, Fletcher et al.^42^ found a benefit in speech understanding scores of 5.3% before and 10.8% after training. CI users, tested with wrist stimulation, had a tactile benefit of 8.3% in speech understanding scores, but only after training^46^.

However, not all studies have shown consistent results, suggesting that the effectiveness of audio-tactile integration may depend on the specific nature of the tactile stimulus. Riecke et al.^51^ did not report any behavioral improvements in speech intelligibility with audio-tactile stimulation compared to audio alone. In contrast to the above-mentioned studies which used features such as the envelope or syllable-timed pulses as the tactile stimulus, Riecke et al. used squared low-frequency portions of extracted frequency-band envelopes, which were entirely complementary to the auditory stimulus. Thus, effective integration of multimodal sensory stimuli may necessitate a degree of redundancy (overlap) between the auditory and tactile signal.

Most recent studies have introduced a control condition, in which the tactile signal is incongruent with the auditory signal. Guilleminot and Reichenbach^48^ included a sham condition in which a tactile pulse train was generated from a different sentence than the audio stimulus. Audio-tactile speech-in-noise comprehension improved by 6.3% over the sham condition and 4.7% over auditory only speech. Similarly, Cieśla et al.^50^ used the tactile stimulus of another sentence as a tactile control in their paradigm, which in this case was a non-matching f0 contour. They replicated an immediate enhancement effect of 4–6 dB difference in SRT consistent with their previous study. Importantly, already prior to training, this effect was present against both the auditory only and the non-matching audio-tactile condition. This enhancement did not improve further, regardless of whether participants received audio-only or audio-tactile training. However, while the difference between the matching audio-tactile condition and audio only did not change, performance in the non-matching audio-tactile condition declined in comparison to audio-only, irrespective of the training type. These findings suggest that training with audio-tactile but also auditory only speech, likely resulting in perceptual learning^52^, seems to be transferable to vibrotactile stimuli. This transfer may have enabled participants to differentiate more effectively between matching and non-matching vibrotactile stimulation resulting in the enhancement effect that remained consistent with matching but was diminished with non-matching tactile stimuli.

These studies have provided valuable insights into the characteristics of stimuli that can enhance audio-tactile speech perception versus those that did not improve intelligibility, probably driven by differences in tactile signal processing used. However, the majority of previous investigations did not incorporate tactile control conditions, leaving room for the possibility of non-specific generally arousing effects influencing the positive outcomes in speech intelligibility.

In light of these considerations, the current study addresses four primary objectives:

1. We studied whether a tactile stimulus based on the speech envelope could improve the ability to understand auditory speech presented in background noise^41,42,45,46,50^.
2. We hypothesized that there would be no significant improvement in speech understanding between auditory-alone performance and performance in a control condition with an incongruent tactile stimulus.
3. We tested this both in NH and CI using a very similar procedure to explore potential differences between the two groups.
4. We assessed whether the PoIE contributed to the results.

## Methods

### Participants

A total of 23 participants with NH and 14 CI users were recruited for this study. One NH participant was excluded due to missing data, resulting in a final sample of N = 22, comprising 10 males and 10 females. NH participants’ ages ranged from 19 to 32, with an average age of 25.7 years (SD = 3.6 years). Among the 14 CI users, 10 were females and 4 were males, with ages ranging from 26 to 71 and an average age of 52 years (SD = 13.5).

### Recruitment

Data collection took place in two different clinics, with the NH sample tested at Copenhagen Hearing and Balance Center (CHBC) at Rigshospitalet, Denmark while CI patients were tested at the German Hearing Center in Hannover using identical equipment. Participants were recruited from the researcher’s social networks, word of mouth and social media. Additionally, CI participants registered in the data base of the German Hearing Center were invited to participate. Recruitment for CI patients was restricted to bilaterally implanted users with at least one year of CI experience on both ears (see Table 1) and a minimum of 50% speech performance in the German Hochmair-Schulz Moser (HSM) sentence test^53^.

**Table 1:** Demographic information of participants using cochlear implants.

Subject	Gender	Age	Onset of hearing impairment^1^	Age of first implantation	Years of CI use	CI brand	Use per day (h)
01	f	48	14	45	3	MED-EL	16
02	f	57	28*	46	11	Cochlear	10
03	m	62	25	57	5	MED-EL	17.5
04	f	54	6	52	2	MED-EL	15
05	f	29	2*	3	26	Cochlear	13
06	f	26	16	23	3	Cochlear	14
07	m	56	9*	36	20	Cochlear	14
08	f	71	18	49	22	Cochlear	10
09	f	58	3*	53	5	Cochlear	14
10	f	58	12	35	23	Advanced bionics	17
11	m	42	8	33	9	Cochlear	13
12	f	65	20*	48	17	Advanced bionics	17
13	m	38	24	31	7	Cochlear	18
14	f	63	26*	52	11	Advanced bionics	15.5

^1^Defined by the age when hearing difficulties were first noticed.

*Onset of hearing aid use was entered when information on subjective onset of hearing loss was missing.

Potential participants received information material regarding the study’s aims, procedures, and their rights as participants before the lab visits. A screening questionnaire, adapted from Fletcher et al.^42^, was administered to identify potential exclusion criteria. All participants were native German speakers and right-handed. Further inclusion criteria ensured the absence of reported psychiatric, cognitive or neurological disorders and intact somatosensory sensitivity on the fingertips. CI patients provided additional information about their hearing loss and CI experience. Five NH participants were excluded due to regular tinnitus symptoms, left-handedness, or previous cerebral bleeding.

### Ethical approval

Ethical approval was obtained from the Danish Science-Ethics Committee (reference H-16036391) for testing of NH subjects at CHBC, and from the Ethics Committee of Hannover Medical School for conducting the same experiment with CI patients at the German Hearing Center. We followed the local study regulations at each site. All participants provided written informed consent prior to data collection.

### Experimental set-up

#### Hardware

Despite the testing occurring in different laboratories for the two experimental groups, the set-up remained identical. Participants were seated in a soundproof booth with a small keyboard positioned at their left hand allowing for responses to questions during the audio-tactile training. Vibrotactile stimuli were delivered via a Bruel&Kjaer mini-shaker type 4810 with a circular contact area of 12 mm diameter, placed under the participant’s right index finger. The mini-shaker was rigidly attached to a heavy steel plate which rested on layers of neoprene foam intended to reduce vibratory coupling and sound generation from the desk. It was connected to a custom-built haptic amplifier. Sound was presented through a loudspeaker (Genelec 8020B) positioned behind the mini-shaker. This positioning aimed to create the perception that both sensory signals originated from the same spatial location. The stimulus file was converted by an RME Fireface UCX audio interface with the audio output channel connected to the loudspeaker and the tactile output signal sent to the haptic amplifier and subsequently to the B&K 4810 mini-shaker.

**Figure 1:**
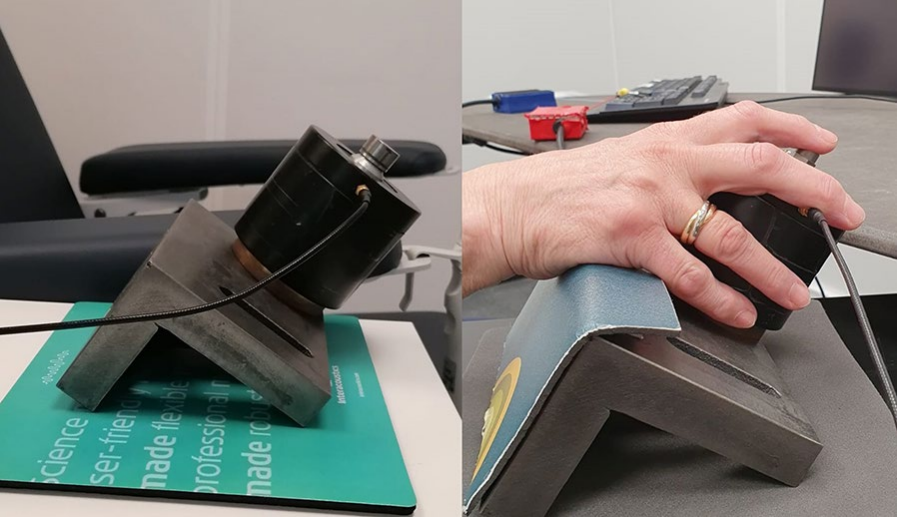
Photographic images showing the tactile actuator. The right picture demonstrates the placement of the right index finger on the mini-shaker.

Additionally, a screen was placed in front of the participant to display visual instructions and provide guidance throughout the experimental blocks, such as for the training period to display questions and solutions. The computer for controlling the experiment was placed outside the booth, where the experimenter could hear the presented stimuli and participants’ answers via a microphone that was placed in the booth. Furthermore, the test person was visible to the experimenter (through a window at CHBC and through a camera at the German Hearing Center) which allowed the experimenter to monitor the test person’s wellbeing and ensure that their hand was positioned correctly on the mini-shaker (see Fig. 1).

#### Software

MATLAB (Version R2020a, Natick, Massachusetts, The MathWorks Inc.) was employed for generating the audio-tactile vocoded sentence stimuli and presenting stimuli for the SRT estimation task and vibrometry measurements. For the main experimental components (audio-tactile training, speech testing) PsychoPy3^54^ was used as stimulus presentation software.

### Stimulus generation

#### Auditory stimuli

The auditory stimuli used throughout the experiment encompassed a variety of speech and musical materials (see Table 2) adapted to an audio-tactile format. For the scored speech testing, the main part of this study, we employed the HSM sentence test material. These sentences contained everyday life content and ranged from three to eight words in length. To reduce audio-alone intelligibility and thus make participants more likely to rely on the available tactile information, speech was presented in a steady noise background, except for one test condition in the CI group in which no noise was added (*easy* condition, see “Speech reception threshold estimation” section below). Unmodulated speech-shaped noise provided with the HSM test was used for this purpose commencing 1.5 s before the speech stimulus for NH participants and 2 s before speech onset for CI users. Speech stimuli were presented at a level of 58 dB SPL LAF for the NH group, while for CI users, the level was adjusted according to individual preference. Speech levels were kept constant with noise added at different levels to target difficulty levels of either 50% or 70.7% intelligibility (for more details, see “Speech reception threshold estimation” section below).

**Table 2:** Stimulus material used for experimental phases.

Experimental part	Stimulus material
Audio-tactile training	
Music	Bass track from the song “szárad a száj,” by Hungarian band “Chalga” Excerpt from Beethoven song Violin Sonata No. 9 “Kreutzer” Amen break Isolation Waltz, public domain song by Bryan Teoh Major scale Instrumental version of “Beat it” by Michael Jackson
Audiobooks	“Wissen macht Ah!–Verblüffende Alltagsphänomene”(Caspers and Reeves^55^) “Frag doch mal… die Maus! Wissen für Kinder: Natur und Geschichte” (Flessner et al.^56^)
Sentence material	Oldenburger Sentence Test (OLSA) (Wagener et al.^57^) Hochmair-Schulz-Moser (HSM) test (Hochmair-Desoyer et al.^53^)
Scored sentence test	Hochmair-Schulz-Moser (HSM) test (Hochmair-Desoyer et al.^53^)

For NH participants, additional distortion of the speech’s spectral properties was applied before adding the noise. Following the processing pipeline established by Fletcher et al.^42^, we used the SPIRAL vocoder^58^. The audio signal was divided into 22 frequency bands with center frequencies equally spaced between 250 and 8000 Hz on the equivalent rectangular bandwidth scale. Eighty random-phase carrier frequencies equally spaced between 300 and 8000 Hz were modulated by a weighted combination of all extracted envelopes, considering a decay slope resembling a current spread of 14 dB per octave. Subsequently, the modulated tonal carriers were summed into a single output signal constituting the vocoded version of the input sentence.

#### Tactile stimuli

##### Congruent tactile stimulus

**Figure 2:**
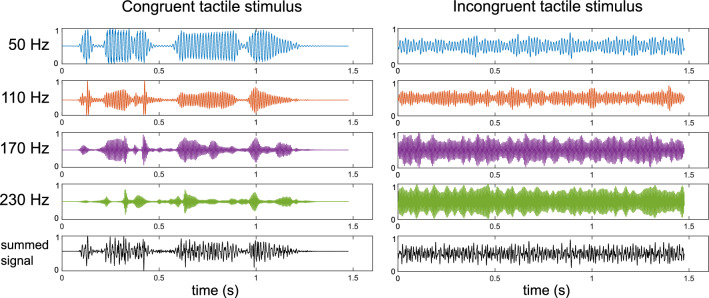
Tactile stimulus synthesis: Left: Envelope modulated carrier-frequencies that are summed to form the congruent tactile stimulus. Right: Incongruent tactile control stimulus consisting of four carriers modulated by unmodulated speech-shaped noise.

A tactile stimulus corresponding to the auditory speech signal was generated similarly to the approach described in Fletcher^46^. However, we created the congruent tactile stimulus based on the clear instead of the noisy speech signal, facilitating the investigation of the enhancement effect per se and its underlying mechanisms. The tactile signal was generated based on four frequency bands spaced between 100 and 1000 Hz extracted from the original audio signal. The envelope of each band modulated a fixed-phase carrier (see Fig. 2). Carrier frequencies were equally spaced between 50 and 230 Hz, corresponding to a frequency range that human skin mechanoreceptors are sensitive to^59,60^. The modulated carriers were then passed through an expander function which attenuated background noise in Fletcher et al.^42^. Here, the effect of the expander is negligible as clear speech was used as the input for the tactile stimulus. Ultimately, the modulated carrier frequencies were summed together and sent to a single haptic actuator for stimulus presentation.

##### Incongruent tactile stimulus

In addition to the congruent tactile stimulus, we generated a tactile signal whose onset and offset was temporally aligned with the auditory speech, but which did not carry any sentence information. This incongruent tactile stimulus consisted of the same carrier frequencies as those used for the congruent stimulus synthesis. Instead of a speech signal, unmodulated speech-shaped noise was utilized as the modulating signal in the pipeline described in the previous section. The resulting stimulus consisted of four noise-modulated carriers summed into one output signal (see Fig. 2).

#### Audio-tactile stimuli for the training phase

The stimuli generated for the audio-tactile training were consistent across all participants within their respective group with fixed SNRs and prechosen vocoding parameters. However, there were differences in stimuli between the two groups.

NH subjects listened to vocoded speech-in-noise with a current spread ranging from − 30 to − 14 dB in SNRs varying between − 6 and 2 dB. This design allowed for the exploration of the constant tactile signal together with the speech signal in slightly varying auditory properties. In contrast, CI patients were exposed to non-vocoded speech-in-noise in SNRs ranging from 0 to 20 dB. Parameters were adjusted for each audiobook chapter and sentence list, gradually increasing the training difficulty. While participants were informed that completing the entire training was not obligatory, they were encouraged to continue until speech became unintelligible. All subjects successfully completed the training.

Regarding musical stimuli, the audio signals were unaltered. The five simple melodies the training started with (see Table 2) had 10 harmonics. Each melody was played twice, once with the tactile signal corresponding to the same melody with only one harmonic and a second time with the tactile signal being generated by processing the 10-harmonic version through the tactile version of the SPIRAL vocoder, as described above. In addition, an instrumental version of popular rock song was used and played through the loudspeaker, with only the bass track transmitted to the mini-shaker. The amplitude of the audio signal was modulated with ramp signals such that the song was faded in and out three times to give the participant the chance of exploring the tactile signal in combination with a varying audio signal (from no sound to full auditory intensity level).

### Procedure

**Figure 3:**
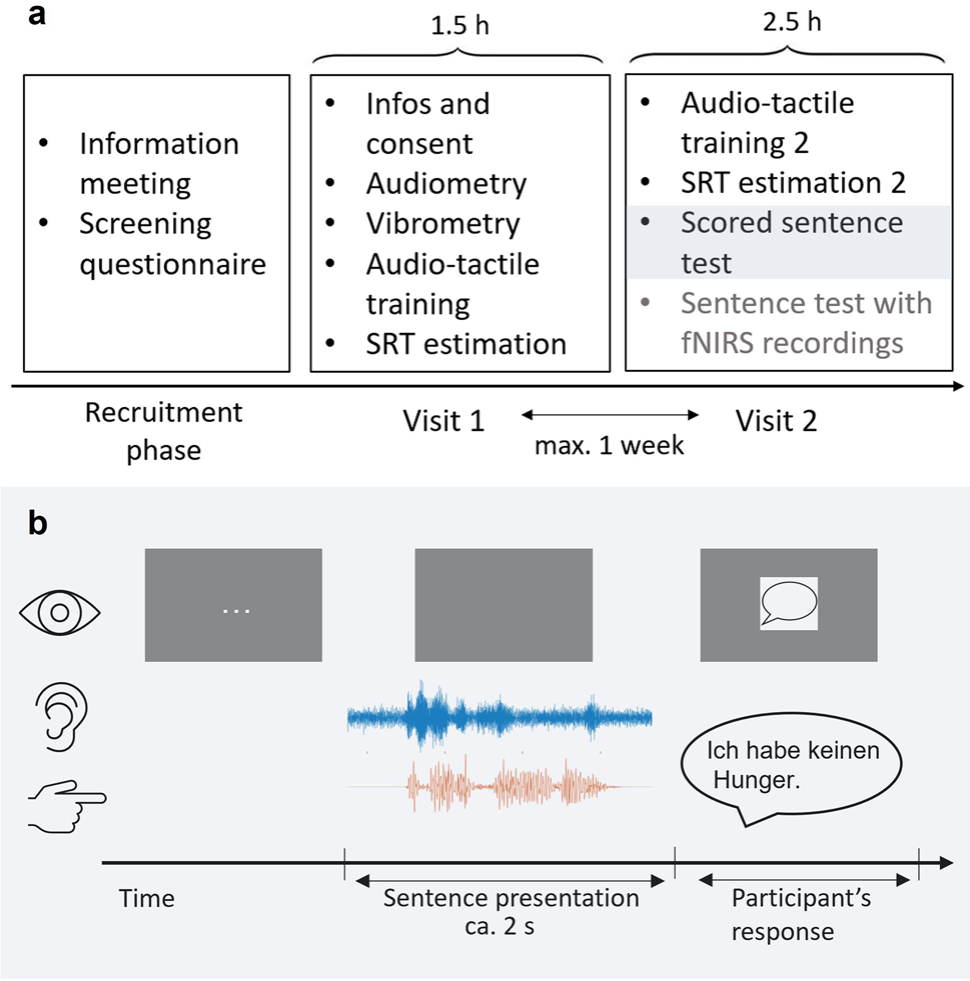
Experimental Procedure. (**a**) Overview of the experimental phases of the study. (**b**) Schematic representation of one trial in a *congruent audio-tactile* condition of the scored sentence test. The sentence “Ich habe keinen Hunger” shown here translates to “I am not hungry”.

Participants underwent two test visits, separated by at most one week. The first visit encompassed screening measures, training, and the estimation of individual speech reception thresholds (SRTs). The first visit of the experiment took approximately 1.5 h. First, participants were informed of the study procedure and their right to end the testing any time. For the CI patients the questionnaire was filled out during the first test visit to allow clarification of questions when needed and conversation about their experiences with their CIs. The second visit involved a shorter training session, repeated SRT estimation, and speech intelligibility testing (see Fig. 3a). In total, visit 2 lasted about 2.5 h. The session included a repetition of the audio-tactile sentence test with concurrent functional near-infrared spectroscopy (fNIRS) recordings. The neuroimaging data will be analyzed as part of another study and not presented here.

#### Audiometry

For the NH group audiometry was performed using Interacoustics Affinity Compact and the Hughson-Westlake procedure^61^. Thresholds were obtained for pure tones at octave frequencies. from 0.25 to 8 kHz. For the NH group all thresholds were < 20 dB HL.

#### Vibrometry

To ensure that participants had sufficient tactile sensitivity on the right fingertip to perceive the stimuli presented during the experiment, we obtained detection thresholds for four sinusoidal tactile signals corresponding to the stimuli’s carrier frequencies (50, 110, 170 and 230 Hz). An adaptive procedure was used with stimulus intensities starting at RMS = 0.1. The level was increased by 5 dB after correct responses and decreased by 10 dB after incorrect responses. All signals were presented with continuous unmodulated speech-shaped background noise at 58 dB SPL LAF via the loudspeaker to mask the sound coming from the shaker. Thresholds were obtained for all four test frequencies consecutively, with 110 Hz tested twice (at the start and end). After obtaining thresholds for each sinusoidal signal, the threshold for a mixed noise signal, composed of all four carrier frequencies added, was determined. This signal corresponds to the incongruent tactile stimulus used as a control condition in the speech test (see “Stimulus generation” section for details).

To determine each subject’s detection thresholds in units of displacement, we measured the acceleration of the actuator for each frequency of interest, transformed this to peak-to-peak displacement in µm, and inferred a linear scale for each frequency. During the experiment, tactile stimuli were sent with an RMS of 0.1. This average presentation level corresponds to a displacement of 17.4, 7.1, 2.8 and 1.4 µm for the carrier frequencies 50, 110, 170 and 230 Hz, respectively. Outcomes of the vibrotactile threshold estimations can be found in the “Results” section.

#### Audio-tactile training

To familiarize participants with combined audio-tactile stimuli and introduce NH participants to the sound of vocoded speech, all subjects underwent two audio-tactile training sessions before participating in the speech intelligibility test. The training encompassed a variety of different speech stimuli including longer excerpts from audiobooks and sentence stimuli from different speech tests spoken by different speakers. A detailed description can be found in the “Stimulus generation” section, and Table 1.

The training was divided into four parts. Part 1 consisted of passive listening to musical stimuli and was designed to familiarize participants with the concept of sound being combined with somatosensory vibrations in general.

Part 2 of the training comprised excerpts of well-known German youth non-fiction audiobooks. These stories covered everyday phenomena and facts about the universe, providing participants with longer and more natural speech exposure than sentence tests, all while using simple language. Excerpts from the chapters were 30–60 s in duration and followed by multiple-choice questions displayed on the screen. Participants answered these using button presses.

To prevent the possibility that the anticipated performance improvement in the *congruent audio-tactile* condition resulted solely from extensive training in that condition, all speech material was alternately played in blocks of *auditory only* or *congruent audio-tactile* conditions throughout the training regime. This was realized by instructing participants to either position their finger on the shaker or remove their finger from the shaker, thereby creating *auditory only* and *congruent audio-tactile* conditions.

Part 3 of the training consisted of 6 lists (10 sentences each) from the Oldenburg Sentence test (OLSA)^57^. To progressively adapt the training closer to the actual speech task format, participants were asked to repeat perceived words aloud after each sentence. Some words from the sentence were displayed on the screen to assist participants. Difficulty increased as more and more words were removed from the screen. After responding to the multiple-choice questions (in part 2) and the OLSA sentences, the correct answer was displayed on the screen.

Part 4 of the training comprised 10 HSM sentences and aimed to familiarize participants with the type of sentences used in the main task. During this last part, the same sentence was repeated four times at increasing SNRs, ranging from − 6 to 9 dB for NH subjects, and from 0 to 18 dB for CI users. Consequently, participants had 4 trials to repeat each sentence correctly.

Training on the second visit comprised a repetition of only parts 3 and 4 with different lists of the OLSA and HSM test used. In total the material used for training over the 2 days adds up to 20 min speech and 7 min music material.

#### Speech reception threshold estimation

Speech intelligibility testing was conducted in an *easy* and a *difficult* condition, with the *difficult* condition targeting 50% intelligibility. For the NH group, individual SNRs were adjusted in the *easy* condition to target 70.7% intelligibility (SRT70.7), while the *easy* condition for CI users did not contain any noise.

To determine the subject’s individual SRT50, we employed an adaptive staircase procedure with a 1 up-1 down progression rule using sentence material from the HSM test. Individual sentences were considered correct if all words were accurately repeated with exceptions for filling words, articles, and grammatical suffixes. After collecting the data with NH participants, we noticed that SRT50s estimated by this rule led to higher performances than 50% words correct. Therefore, for testing the second participant group, the scoring rule was adjusted to SRTs being decreased as soon as two thirds of the sentence were reported correctly.

The step sizes for the procedure were 4 dB for the first 8 sentences (one third of the sentence set) and 2 dB for the remaining 14 items, totaling 22 sentences for one run. The first sentence was presented at 4 dB SNR for CI users and 8 dB SNR for NH subjects. NH participants were exposed to vocoded-speech-in-noise (for more information on the vocoder and noise properties see the “Stimulus generation” section), while CI patients were presented with non-vocoded speech-in-noise. The SRT was defined as the mean of the SNRs at the last 6 reversals.

This procedure was repeated four times on new stimulus material (twice in each visit), resulting in a total of four SRT50 estimates, which were then averaged and rounded to the nearest integer. For NH subjects SRT70.7 was inferred by adding 4.5 dB to the average of the four SRT50 estimations and subsequently rounding to the nearest whole number. The estimated difference of 4.5 dB between SNRs for both difficulty levels was based on the results of SRT70.7 estimations in seven pilot subjects using a 1 up-2 down procedure^62^ which was conducted over two rounds of 35 test items.

Within the NH group, the mean SRT was − 5.74 dB SNR for 50% intelligibility, and − 1.40 dB SNR for 70.7% intelligibility. In the CI group the mean SRT50 was − 1.71 dB SNR.

#### Speech intelligibility testing

Speech intelligibility was compared across six different test conditions: *Auditory only, congruent* and *incongruent audio-tactile* conditions, each presented at two difficulty levels (*difficult *(SNR50) and *easy* (SNR70.7) for NH participants, *difficult* (SNR50) and speech without noise as the *easy* condition for CI users). Stimuli were grouped by condition and presented in blocks of 20 sentences per condition, resulting in a total of 120 sentences for the behavioral speech intelligibility test (for an example of a trial in a *congruent audio-tactile* condition, refer to Fig. 3b). For each condition, word scores of five additional sentences were obtained during the fNIRS speech test (fNIRS data is not analyzed here). These scores were considered together with scores of the behavioral part, resulting in a total of 25 sentences for each condition. The number of words varied across the sentences but was balanced over lists, such that the 25 sentences correspond to 131 words for each condition. The speech intelligibility score for each condition was calculated as the percentage of correctly repeated words out of 131.

To eliminate any auditory advantage resulting from audible sound produced by the mini-shaker in the audio-tactile conditions, the congruent tactile signal was also played during *auditory only* conditions, although the participants’ finger was not touching the mini-shaker. Clear instructions were provided to participants at the beginning of each block to either place their finger on the shaker or remove their finger from it. This way it was ensured that the participant was presented with the correct condition with compliance additionally observed through the camera system (at German Hearing Center) or observation window (CHBC). Furthermore, *audio-tactile* blocks were introduced with the message “Tactile enhancement ON, audio enhancement OFF”, and *auditory only* blocks with “Tactile enhancement OFF, audio enhancement ON”. This approach was employed aiming to prevent that participants did not anticipate better performance in any specific block, as they received the impression that all blocks were enhanced in different ways.

The order of condition blocks was balanced among subjects with one constraint: the two *auditory only* conditions were equally spaced between the other conditions to allow breaks between prolonged tactile stimulation. A Latin square was generated to determine possible condition orders, with *auditory only easy* and *auditory only difficult* conditions fixed at all possible position combinations, ensuring that they did not follow each other and were not presented as first or last condition block. This resulted in 24 condition combinations, such that each participant was tested with a different condition order. Sentence material was consistent across all participants, meaning that the same sentences were presented in the same order for all subjects, but they were presented as either *congruent* or *incongruent* audio-tactile conditions in either the *difficult* or *easy* condition.

Upon completing the experiment, each participant was asked about their subjective experience with the task, specifically whether they found the tactile support helpful and whether they perceived a difference between tactile stimuli.

### Statistical analysis

Before conducting our statistical analyses, we visually explored the results of the speech intelligibility test. Both the NH and CI groups exhibited ceiling effects in all *easy* task conditions (Fig. 5a). Testing the presence of the PoIE by the hypothesis of a larger enhancement among difficult than among *easy* conditions, as initially planned and recommended by the literature^24^ was therefore not meaningful. Instead, we limited all further statistical analysis to results of the *difficult* conditions only. To test our hypotheses of performance enhancements in *congruent audio-tactile* conditions over *auditory only*, and over *incongruent audio-tactile* conditions, but no enhancement for *incongruent audio-tactile* over *auditory only* conditions*,* we conducted three one-sided paired t-tests. To account for non-linear performance changes, the analysis was repeated on logit transformed test scores. Results were Bonferroni-corrected for multiple comparisons within each group. Shapiro–Wilk tests indicated no deviation from normality in the difference in word scores between the planned comparisons in either group. Finally, a two-sided independent t-test was performed to investigate potential differences in the size of the enhancement effect (defined as *congruent audio-tactile*–*auditory only* performance) between NH and CI participants.

Further, we employed Pearson’s correlations to assess potential interactions between participants’ *auditory only* performance and their tactile benefit within each group. Although correlation analyses is a commonly applied approach to assess inverse effectiveness^47,63^, its problem of shared variances between both variables is sometimes overlooked and not corrected for^13,24^. To address this issue of regression to the mean, we tested the observed correlations against a null distribution of correlations illustrating the dependencies in the data. With 10,000 repetitions, we resampled the data with replacement from *auditory only* and *congruent audio-tactile* conditions separately (bootstrapping), followed by calculation of the Pearson’s correlation.

Additionally, we aimed to understand whether participants subjectively perceived that they understood more words with the support of a tactile signal and whether this perception correlated with their test outcomes. We examined whether the average tactile benefit was higher among individuals who found the stimulus helpful, employing a Student’s t-test. Furthermore, we used Fisher’s exact test, considering the odds ratios to investigate whether a positive tactile benefit was associated with finding the stimulus helpful. A related question addressed whether participants discern a difference between the *congruent* and *incongruent audio-tactile* condition. We conducted similar statistical testing (t-test and Fisher’s exact test) considering participants test performances and answers to this question. Lastly, we used Spearman’s rank correlation to test for potential relationships between age, vibrotactile detection thresholds and tactile benefit (non-normality confirmed by Shapiro–Wilk tests for age and vibrotactile thresholds).

All described statistical analyses and figures in this manuscript were created using R^64^.

## Results

### Vibrometry

**Figure 4:**
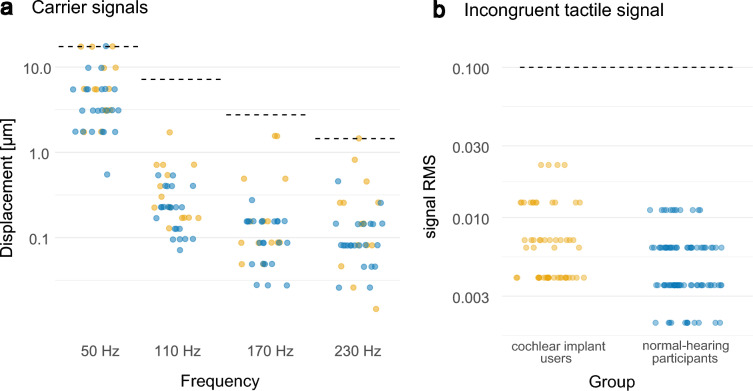
Vibrometry thresholds. (**a**) Participants’ individual vibrotactile detection thresholds for the four different carrier frequencies, displayed on a logarithmic displacement scale in μm. Cochlear implant users are plotted in yellow, normal-hearing participants in blue. (**b**) Estimated detection thresholds for the incongruent tactile signal (consisting of all four frequencies mixed) displayed with the tactile signal amplitude (rms of signal waveform). During speech testing, all signals were sent with an rms of 0.1, indicated by the dashed lines.

Vibrotactile detection thresholds on the participants’ right fingertip were estimated to ensure that the presented stimuli during training and speech intelligibility testing were perceivable for the subjects (see Fig. 4).

Mean detection thresholds over the four vibrotactile frequencies tested for CI users were significantly higher than for the NH participants (mean difference 133.3%, U = 97, *p* = 0.046). This was also the case for the incongruent tactile signal (mean difference 150.2%, U = 3840, *p* < 0.01). The latter mentioned thresholds estimates increased with age, taken both participants groups together (r(34) = 0.51 *p* = 0.001).

During the task, tactile stimuli were presented at a signal level of RMS = 0.1 and were thus over threshold for all participants. We verified that the entire dynamic range of the tactile speech signal (which was on average 38 dB) lay within the perceptual range by testing that there was no physical saturation of the mini-shaker for the maximal displacements in the tactile stimulus.

### Speech intelligibility

**Figure 5:**
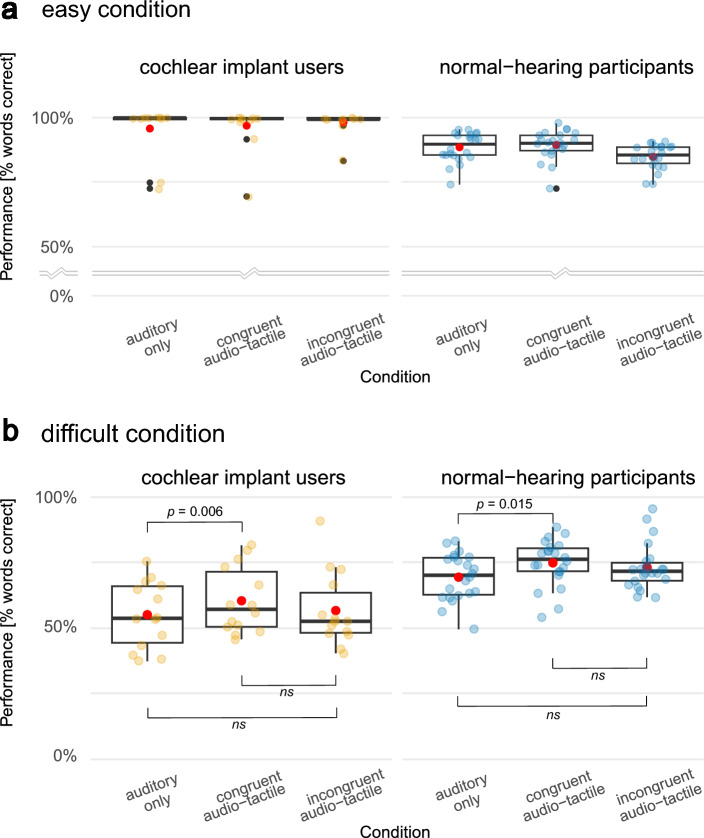
Speech intelligibility test results. Scores of the HSM sentence test are displayed in % words correct, with mean scores of each condition indicated by a red dot. Data plotted in yellow represents cochlear implant users and data plotted in blue the normal-hearing participant group. (**a**) Results for the *easy* task conditions. For normal-hearing listeners, stimuli in the *easy* condition were played at their individual SRT50 + 4.5 dB SNR, while for cochlear implant users no noise was added. (**b**) Results for the *difficult* condition. Note that due to ceiling effects in the *easy* condition, only the *difficult* condition was considered for statistical analysis.

Speech intelligibility testing for the *difficult* condition revealed that the presence of a tactile envelope signal (*congruent* audio-tactile speech) enhanced speech intelligibility compared to *auditory only* sentences in both groups (Fig. 5b). For the NH group, the mean test performance was 5.4% higher in the *congruent audio-tactile* compared to *auditory only* condition (*t*(21) = 2.66, *p* = 0.015). For CI patients this difference (= tactile benefit) was 5.3% (*t*(13) = 3.3, *p* = 0.006). In the *incongruent audio-tactile* condition the mean increase in performance was *3.7*% in NH test participants (ns) and 1.62% in the CI group (ns) compared to *auditory only* condition. There was no significant improvement of performances in the *congruent audio-tactile* compared to the *incongruent audio-tactile* condition in either the NH group (*t*(21) = 0.86, *p* = 0.2, or CI group *t*(13) = 1.54 *p* = 0.07). The pattern of results remained identical when performing these comparisons on logit transformed values (see Fig. S1).

#### Individual tactile enhancements

Besides comparing the group means, we examined the difference between *congruent audio-tactile* and *auditory only* performance for each subject and used these values to compare the audio-tactile enhancement effect between the two groups.

**Figure 6:**
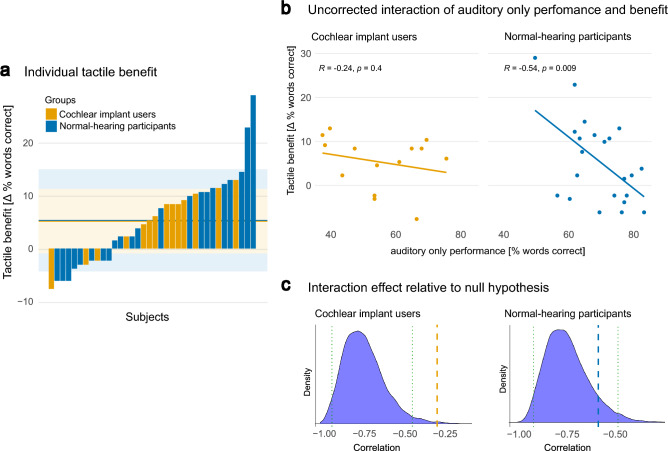
(**a**) Individual tactile benefit (defined by *congruent audio-tactile*–*auditory only* task performance in % words correct) for each participant. Bars in yellow represent normal-hearing participants, bars colored in blue represent cochlear implant users. The mean tactile benefit for each group (5.3% for cochlear implant users and 5.4% for normal-hearing participants) is indicated by the solid line, with the shaded area representing the standard deviation. (**b**) Interaction of *auditory only* performance and tactile benefit as defined by a Person’s correlation, not correcting for dependencies between the variables. (**c**) Bootstrapped correlation density plots. The density of Pearson correlation coefficients obtained from 10,000 bootstrap resamples is displayed, illustrating the likelihood of obtaining a certain correlation between the auditory only and tactile benefit by chance. 95% confidence intervals are indicated by the dotted green lines. Dashed lines mark the observed correlation coefficient shown in (**b**), demonstrating that for cochlear implant users, there was a significant positive correlation relative to the null distribution of correlations (left). For normal-hearing participants the observed coefficient is not significantly different than the random variation inherent in the data set (right).

Figure 6a shows the individual benefit for all participants ordered by the size of the enhancement as well as the mean tactile benefit for each group. What is striking is the extremely high benefit in two of the NH sample. Three out of 14 CI users (21.4%) performed worse when receiving the simultaneous tactile signal in addition to the auditory stimulus. Among the NH participants, this ratio was higher with 8 out of 22 participants (36.4%). As expected from the values, the mean tactile benefit of 5.3% and 5.4%, were not statistically different between the two groups (*t*(34) = 0.04, *p* = 0.97).

According to the PoIE, we expected the tactile benefit to increase with decreasing *auditory only* performance. When not correcting for the shared variance present between the *auditory only* and tactile benefit (*congruent audio-tactile* minus *auditory only*) variables^24^, the expected negative correlation was present in the NH group (*r*(20) =  − 0.54, *p* = 0.009), while it was not significant in the CI group (*r*(12) =  − 0.24, *p* = 0.4) (Fig. 6b).

However, taking the bias towards a negative correlation into account and comparing the observed correlations against the data’s null distribution of correlations as identified by bootstrapping, the results were different: For the NH group, the bootstrapping procedure resulted in a null distribution with a mean correlation of *r*(20) =  − 0.7, indicating the correlation coefficient to be expected by chance. The observed correlation of *r*(20) =  − 0.54 lies within the 95% confidence interval (− 0.88 to − 0.44) of the bootstrapped null distribution of correlations, and thus indicates that the level of audio tactile benefit is not significantly correlated with the *auditory only* performance (*p* = 0.09). For the CI group, the observed correlation of *r*(20) =  − 0.24 is more positive than the mean of the generated null distribution (*p* = 0.005, *r*(20) =  − 0.7, confidence interval =  − 0.9 to − 0.4), indicating a significant positive relationship between *auditory only* scores and the tactile benefit (see Fig. 6c).

### Subjective enhancements

Further exploratory analysis investigated whether speech intelligibility scores related to the subject’s subjective experiences with the tactile stimulus. Thirteen out of the 22 subjects with typical hearing (59.1%) reported that they found the tactile support helpful (3 abstentions), while only 5 out of 14 (35.7%) CI users found the tactile support helpful (4 abstentions). The mean tactile benefit of participants who *did* subjectively perceive an enhancement was 4.8% larger than in those who reported no subjective enhancement (ns). Fisher’s exact test confirmed a non-significant tendency of those participants finding the stimulus helpful receiving a benefit in speech understanding from the audio-tactile stimulus (*p* = 0.21).

In addition, participants were asked whether they perceived a difference between tactile stimuli. 13.6% (5 abstentions) of the NH and 28.6% (4 abstentions) of the CI group noticed that there were differences between tactile conditions. Mean tactile benefits were not larger in participants who noticed a difference (*t*(31) = 1.63, *p* = 0.11), confirmed by an absent association between both variables (Fisher’s exact test (*p* = 0.16)).

#### Other contributing factors

Spearman’s rank correlations were computed to test for potential relationships between the amount of tactile benefit and other possible contributing factors: Age, vibrotactile detection thresholds and the time between the first and second testing sessions. None of these correlations were significant.

## Discussion

When the haptic signals were *congruent* with the auditory stimuli, we observed improved speech understanding scores in both CI users (by 5.4% over *auditory only* performance) and listeners with NH (by 5.3% over *auditory only* performance). Importantly, there was no significant speech understanding benefit when the tactile signal was *incongruent* with the auditory signal. However, we did not show a significant tactile benefit for *congruent audio-tactile* speech relative to the *incongruent audio-tactile* condition. Additionally, participants’ subjective awareness of congruent or incongruent tactile stimuli during the experiments, as well as their subjective rating of the effectiveness of tactile support were not associated with the objective test outcome (tactile benefit). This suggests that a placebo effect or self-fulfilling prophecy, driven by expectations of (congruent) tactile stimuli enhancing speech intelligibility, can be ruled out as an explanation for the result.

In both participant groups, we replicated a tactile enhancement effect for a congruent audio-tactile stimulus as reported in most previous investigations^42,45,46,48,50^ Our effect size appears to be smaller than in the other studies, although a direct comparison to scores reported in SNR reductions^41,45,47,50^ is not possible with the psychometric curves for each study’s participant group and test material unknown. In general, due to small sample sizes and an interaction of factors that were different between investigations (multi-talker vs. stationary background noise, stimulation to the wrist vs. finger), variation in the reported benefits can be expected. Nevertheless, we aim to infer potential patterns of successful audio-tactile enhancement, by the comparison of ours to previous work with a focus on the studies by Fletcher et al.^42,46^ whose study protocol and tactile signal processing was closest to ours.

### Stimulus difficulty

Overall performance between the studies appear to be quite similar (scores ranging from 61.5 to 72.3% (NH) and 54.3–62.5% (CI) in Fletcher’s studies, here it ranged from 69.5 to 75.0% for NH and 55.18–60.5% for the CI group), so differences in stimulus difficulty are unlikely to be a explanation for the smaller enhancement we found (5.3% compared to 8.3% in CI users, and 5.4% compared to 10.8% in NH listeners) compared to Fletcher et al.^42,46^.

It is worth noting that in both investigations, mean scores were consistently higher in the NH groups than in the CI groups. When purely considering the performance level and neglecting other differences between groups, the PoIE would suggest a greater tactile benefit among the CI users, which was neither present here nor in the previous investigations^42,46^. In our study this group difference in overall performance levels can partly be explained by the less strict decision rule used during the SRT estimation procedure for the CI group. Nevertheless, as this phenomenon is also present in previously reported results, there might be additional differences between the groups in maintaining the targeted SRT50 performance throughout the task. In both cases the CI group was significantly older than the NH group which could explain differences in cognitive abilities such as sustained attention^65^, processing speed^66^ and memory acquisition and retrieval^67^. Older participants may experience declines in task performance over time, while younger participants may improve with training throughout the task. Ideally, the NH control group would be matched in terms of age and cognitive function to increase the likelihood of similar performance levels between groups.

### Efficacy of audio-tactile training

The pure amount of training is unlikely to explain the variation in effect size between our study and Fletcher et al.^42,46^. While Fletcher et al.^42^ included 30 min of speech material distributed over three sessions in their training program, we used 20 min of speech material and 7 min of musical stimuli. However, the training duration in the *congruent audio-tactile* condition, specifically, was lower because half of the time, participants were trained in the *auditory only* condition to rule out an enhancement effect due to condition-specific training. Thus, speech in the *congruent audio-tactile* condition was only trained on 10 min of material in this study which is one third compared to Fletcher et al.^42^ and half of the time participants spent with audio-tactile speech in Fletcher et al.^46^. Although similar outcomes after auditory only and auditory-tactile training in Cieśla et al.^50^ suggest that the differences in test results due to our training with mixed conditions compared to purely audio-tactile training are insignificant, the effects seen might be specifically resulting from training on the stimulus material from the same sentence test as used for speech intelligibility testing. This transfer effect between modalities is most likely due to perceptual learning^52^ and might be material specific and influenced by the fact that the training and testing material was recorded by the same speaker^68^. Finally, even with different speakers, training tasks performed closer to the actual test (repetition of single sentences) and the same training time spread out over three days may have led to larger tactile enhancements in Fletcher et al.^42,46^ than in our study.

However, the effectiveness of training in this case is evaluated with respect to a sentence test, raising questions about ecological validity. In our study, we did not record test scores prior to training, and hence, we cannot draw inferences of the effectiveness of the training on our test outcome directly. However, compared to previous studies, we included a much larger variety of stimuli, including also musical stimuli, pieces of more natural speech from audiobooks, followed by a small variety of tasks rather than invariably asking participants to repeat the previous sentence. For audio-tactile speech to have potential future application in real life, it is important to show that training is generalizable, such that participants can experience improvements of intelligibility for novel stimuli. In this aspect the training in the present study was closer to realistic condition than previous investigations. Nonetheless, we see room for further improvements by incorporating more interactive and engaging training approaches. For instance, participants could be provided with the option to select training material from a variety of podcasts, creating a more personalized and immersive experience. Further, we could integrate gamification elements into the training regimen, involving tasks like matching sounds or sentences with their corresponding tactile signals. As well as enhancing the enjoyment of training, it could also encourage participants to actively explore the tactile signals, potentially leading to even more effective tactile enhancements.

### Is redundancy between the auditory and tactile signal related to effective enhancement?

Besides small differences in training, one factor that may have contributed to the discrepancy in effect size is the difference in how the tactile signals were generated. Fletcher et al.^40,41^ derived the tactile stimulus from noisy speech, whereas in our study we used clear speech and added noise only to the auditory stimulus part. Surprisingly, this did not hinder the tactile enhancement but, conversely, triggered an even larger tactile benefit. Possibly, including noise in the tactile signal leads to a greater perceived overlap between the two signals. This increased similarity might facilitate sensory integration, ultimately enhancing speech intelligibility. If this hypothesis holds true, it could be advantageous for the use of tactile aids in natural environments and real-time processing, where a noisy tactile signal would be much easier to extract than a clear one.

The degree of redundancy between sensory signals may also be related to the presence of audio-tactile enhancements in most studies and the absence of a behavioral benefit observed in Riecke et al.^51^. Previous research has demonstrated that the similarity of stimuli influences the tendency for integration, as has been shown behaviorally^69,70^ and supported by neural evidence in the superior colliculus^31^. To delve deeper into this aspect, a closer analysis of the redundancy level between stimuli in the different studies is essential. Such analysis could quantify the redundancy by an objective metric and investigate the optimal overlap between auditory and tactile stimuli. Understanding what tactile features are most effective to be presented through the somatosensory modality is crucial for optimizing tactile stimulation and, consequently, shaping the effectiveness of multisensory aids to speech understanding.

### The content of the tactile stimulus matters for task performance

Guilleminot and Reichenbach^48^ reported a similar enhancement effect to our result (4.7% improvement in mean speech understanding scores), despite having lower overall speech intelligibility scores (45.3–50%). In contrast to our findings, they observed an even larger enhancement (6.3%) when compared to an incongruent tactile stimulus^48^. A similar pattern was also observed in the investigation by Cieśla et al.^50^. In both studies, the incongruent tactile signal consisted of another sentence’s tactile signal, which seemed to hinder successful and accurate perception of the presented sentence rather than enhancing it (as with the trend observed in our study). Although all sentence material shared a very similar syntactic pattern and had little variation in lengths, these studies demonstrated that non-matching amplitude fluctuations in the tactile signal interfered with auditory speech perception and had a negative impact on speech intelligibility.

In our study, we selected a tactile control condition consisting of stationary noise with the same duration as the auditory signal. Although this signal lacked speech information, its onset and offsets were temporally aligned with the auditory stimuli, thereby providing a temporal cue about the beginning (and end) of the sentence. Interestingly, the observed pattern of mean performance across our three conditions corresponded to the amount of information conveyed through the tactile domain: no additional cues in *auditory only* stimuli, a sentence onset cue in * incongruent audio-tactile* sentences, and both an onset cue and envelope fluctuations in the *congruent audio-tactile* condition. While test performance in the *incongruent audio-tactile* condition did not show a significant improvement over *auditory only* performance, all three conditions considered together reveals a stepwise pattern influenced by the overall informational content. It is likely that the temporal information about energy changes of the speech signal facilitated parsing of the sentence and hence enhanced intelligibility. Whether this enhancement resulted from an integrative process or was rather driven by a top-down attentional enhancement requires a closer look at individual scores beyond the group effect and potentially additional research involving measurements of neural activity at different stages of speech processing.

### Similar enhancement effects in both groups: a result of successful multisensory integration?

Controlling for difficulty levels, we found no difference in tactile enhancement between the two groups, indicating generally similar MSI abilities in our samples. However, it does not automatically follow that the observed enhancement effect was due to true neural integration of the two unisensory signals. To address this question, we considered whether our results aligned with the principles of MSI. Typically, integration is assumed to have occurred when responses to congruent stimuli show multisensory enhancements that are larger than the sum of unisensory responses (super additivity^14^) and increase for less reliable unisensory stimuli (PoIE). However, several concerns have been raised with respect to whether these principles (initially defined to account for firing behavior of single cells) are transferable to other neuroimaging techniques and behavioral measures^14,24^. For audio-tactile speech intelligibility paradigms, super additivity is not even assessable, because no intelligibility can be expected from tactile cues alone.

Testing for the presence of the PoIE, we investigated how the size of the tactile benefit might be different for individual participants, based on their auditory alone performance. Given the problem of regression to the mean inherent to a correlation analysis between *auditory only* performance and the tactile benefits^13,24,71^, we tested the hypothesized effect against a null hypothesis that reflects the bias towards a negative relationship in the dataset. In the NH group, our findings indicated that the observed correlation did not significantly differ from what would be expected by chance. In the CI group, the bootstrapping analysis revealed the opposite than expected effect, that is increasing tactile benefits with increasing *auditory only* speech intelligibility. Instead of pointing towards a tactile benefit that can be explained by MSI, these results suggest that the tactile signal can be used more effectively when *auditory only* performance is better. This could be explained by shared attentional resources that are needed to consider tactile stimuli as a potential source of information. Possibly, those participants who struggled more with the task (lower *auditory only* performance), tried to focus on the auditory signal and had less remaining capacity to pay attention to the tactile stimulus. On the other hand, participants with good *auditory only* performances may have had the attentional resources to explore also other sources of information, in which case the tactile signal enhanced speech intelligibility further. This also implies that directing at least some attention towards the uncommon tactile stimulus might be a prerequisite for enhancements taking place. Potentially, participants with poorer performance require more time and training to familiarize themselves with this divided attention task.

Overall, these conclusions are made based on intelligibility levels of around 50%, neglecting differences in listening performance and strategies employed in everyday life. Compared to the literature, the mean performance of our CI sample with 55% words correct at − 1.71 dB SNR, appears to be substantially above average (e.g. mean scores of 0% words correct at SNR 0 dB^53^ and 48.5% at an SNR of 10 dB^72^). Therefore, for the CI group, it needs to be considered that all analyses are based on a sample of participants that probably does not well resemble the population's range of speech intelligibility performance. It might be that the positive relationship of effectiveness in our well-performing group of CI users is not generalizable to poorer performing CI users, who are less auditorily skilled and potentially more used to employing multisensory listening strategies. To draw valid conclusions about the presence of inverse effectiveness, a larger group of participants with a wider range of performance levels is required. From our data set alone, it is not possible to infer definite claims about whether audio-tactile speech enhancement generally follows the PoIE and whether true multisensory integration constitutes the underlying mechanism of the observed enhancement effect, but in the present sample PoIE was not observed.

### Potential benefits beyond speech intelligibility enhancement

The use of tactile aids may offer benefits beyond enhancing speech intelligibility, such as reductions in cognitive effort. High listening effort in CI users has been described both subjectively and objectively in behavioral measures^73^ and listening fatigue is a great problem for many CI users impacting quality of life^3^. Objective neural measurements have shown evidence of increased recruitment of cognitive resources, as indicated by frontal lobe activity in CI users compared to NH controls^74^. This suggests that CI users may have fewer cognitive resources available for other concurrent tasks.

The possibility that combined audio-tactile speech might facilitate listening by reducing cognitive effort, independently of its impact on intelligibility tasks, has not been investigated thus far. If this hypothesis proves to be true, a tactile aid could help listeners sustain their attention for extended periods and alleviate listening fatigue. Furthermore, the fact that Cieśla et al.^45,50^ demonstrated the most substantial improvements for non-native listeners, suggests that vibrotactile support may offer benefits when listening to foreign speech or for language learning. Remarkably, the effect was observed without any training and for stimuli that can be assumed to have little cross-modal redundancy (f0-envelopes as the tactile signal and vocoded speech as the auditory signal). This highlights that the principles such as improvements by training and redundancy facilitating integration, which seem to hold for investigations on native speakers^42,46,48^ may not apply in the same way for listening to non-native speech. An fMRI study demonstrated that neural activation associated with syntactic processing differs between native and non-native languages^75^. The authors found elevated activity in frontotemporal language networks among non-native listeners indicating an increased cognitive demand for speech parsing. This finding, together with our hypothesis that facilitation of speech parsing drives the observed tactile benefits, constitutes a possible explanation for the differences in effect size in tactile intelligibility improvements between native and non-native listeners. Non-native participants face intelligibility challenges not only due to noise masking but also due to their more limited language experience. In contrast, native participant groups might decode syntactic structures already more effectively from the auditory signal alone, reducing their reliance on tactile support.

Building on this concept, anyone not perfectly accustomed to certain auditory speech inputs might make most efficient use of vibrotactile cues. This principle could extend to newly implanted CI users, who typically undergo intensive training to map the new auditory percepts to their corresponding linguistic element. During this critical phase of (re)habilitation, the support of a tactile stimulus could be advantageous in focusing on the syntactic structure of sentences, thereby facilitating the interpretation of newly perceived sounds as distinct syllables and words.

### Outlook

Already with the current knowledge, we see potential for tactile aids to become valuable tools for improving communication abilities for CI users, especially in cases where the intelligibility of unimodal speech is unsatisfactory and demanding, and for initial rehabilitation with the CI. It is encouraging to note that speech intelligibility can improve after brief training periods^42,46,50^. In real-world applications of tactile support, individuals would interact with the device more frequently and in a wider range of scenarios than possible to test in a controlled study. Once an audio-tactile application is established in everyday use, regular exposure and training are likely to yield increasing benefits in speech intelligibility and, potentially, reductions in cognitive effort.

### Limitations

This study has several limitations that should be considered, with some of them pointed out earlier during the discussion. For one thing, age was different between both participant groups. Vibrotactile sensitivity has been shown to decrease with age^76,77^ which is consistent with our findings. The constant stimulus intensity during speech testing may not have been as salient for many CI users, as it was close to their average threshold. Lower vibrotactile thresholds have been reported for congenitally deaf individuals^78^, leading to the hypothesis that the CI group would have better sensitivity if they were of the same age as the NH sample. This effect, if present for a not fully congenitally deaf group with restored hearing, may be masked by the age effect. The detection threshold difference may thus be more likely due to the age difference. Importantly, neither age nor vibrotactile detection thresholds correlated with the tactile benefit, so that contribution of these factors to the results can be ruled out.

Finally, although we included *auditory only* speech as a training condition, we cannot rule out potential training-specific effects compared to the *incongruent audio-tactile* condition, which was not part of the training regime. Test performance might have been slightly different if we had included training on all three conditions. Further research with an equal amount of training in all test conditions is needed to ascertain whether the conclusions of our and previous studies, stating that only *congruent* tactile signals enhance speech intelligibility, persists.

## Conclusion

In summary, this study provides evidence that vibrotactile stimuli can improve speech intelligibility in challenging listening scenarios if they are congruent with the speech signal. Presumably, temporal information provided by the congruent vibrotactile stimulus cues speech segmentation and thereby enhances intelligibility. CI users were found to benefit equally well as NH participants. Within the highly auditorily skilled group of CI users tested in this study, enhancement increased with better auditory task performance, potentially resembling the use of an auditory-focused listening strategy. Contrary to our expectations, these results challenge the principle of inverse effectiveness and question its application to behavioral data. We hypothesize that in addition to the high-performing CI users investigated in this study, anyone struggling with auditory only speech-in-noise intelligibility could significantly benefit from vibrotactile support. Particularly during the rehabilitation of newly implanted adult CI users, vibrotactile speech cues may effectively support the recognition of syntactic sentence structure from the new auditory percepts.

### Supplementary Information

Supplementary Figure S1.

## Data Availability

The datasets generated analyzed during the current study are not publicly available due to data protection issues but are available from the corresponding author on reasonable request.
