# Supplementary material for: Improved speech intelligibility in the presence of congruent vibrotactile speech input
Source: Sci Rep. 2023 Dec 19;13:22657. doi: 10.1038/s41598-023-48893-w (PMC10730903; doi:10.1038/s41598-023-48893-w)

**Improved speech intelligibility in the presence of congruent vibrotactile speech input**

Alina Schulte*^1,3^, Jeremy Marozeau^2^, Anna Ruhe^3^, Andreas Büchner^3^, Andrej Kral^3^, Hamish Innes-Brown^1,4^

^1^Eriksholm Research Center, Oticon A/S, Snekkersten, Denmark

^2^Music and Cochlear Implants Lab, Department of Health Technology, Technical University of Denmark, Denmark

^3^Institute for AudioNeuroTechnology (VIANNA) & Dept. of Experimental Otology of the Clinics of Otolaryngology, Hannover Medical School, Hannover, Germany

^4^ Hearing Systems Section, Department of Health Technology, Technical University of Denmark, Denmark.

# **Supplementary information**

**Figure S1**. Logit transformed speech intelligibility test scores for the *difficult* condition. The results displayed here are equivalent to Figure 5b. Scores on the y-axis in percentage words correct were transformed into the log of their odds ratio. As a result, 50% performance corresponds to a logit of 0, with a scale ranging from minus infinity to infinity. Extreme ends of the percentage scale are “stretched”, such that performance differences between very high or very low percentages are more pronounced. Means in each condition are plotted with a red dot. Data plotted in yellow represents cochlear implant (CI) users and data plotted in blue the normal-hearing (NH) participant group. Repeating the statistical analysis on logit transformed percentages led to the same conclusions as in our original analysis. NH group: Mean scores of the *congruent* audio-tactile condition were significantly larger than *auditory only* scores (*t*(21)=2.77, *p*=0.01), while those of the *incongruent* audio-tactile condition were not (*t*(21)=2.17, *p*=0.06). There was no difference between *incongruent* and *congruent* audio-tactile scores (*W*(21)=147, *p* =0.7. The same comparisons within the CI group yielded the same pattern of results: *t*(13)=3.43, *p*=0.007 (*congruent* vs. *auditory only*) , *t*(13)=0.78, *p*=0.7 , (*incongruent* vs. *auditory only*) and *t*(13)=1.14, *p*=0.8 (*congruent* vs. *incongruent*). All p-values were Bonferroni-corrected.


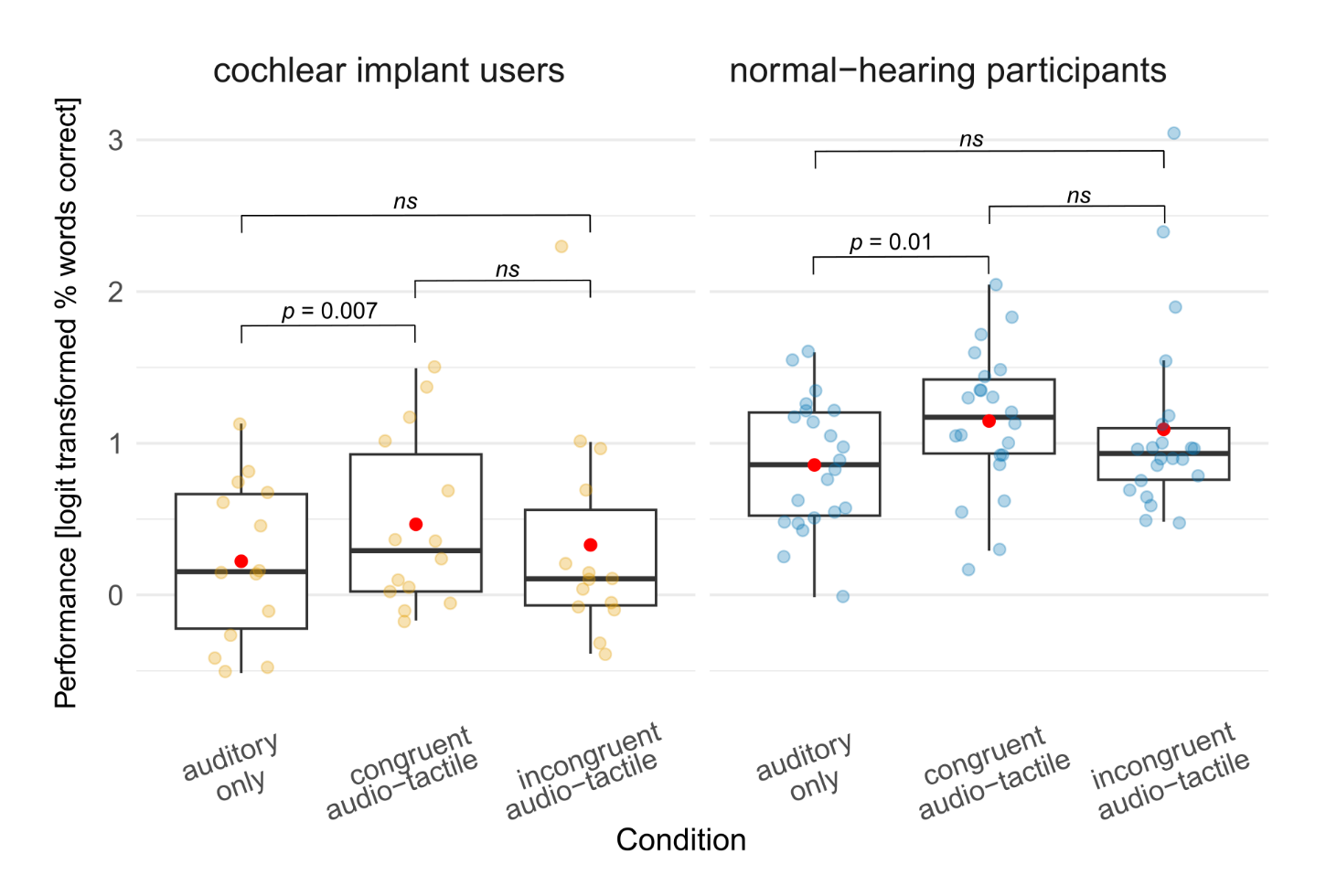

Supplement: Supplementary file 1 — Supplementary Figure S1. [file 41598_2023_48893_MOESM1_ESM.docx]
